# A conceptual framework: the early and late phases of skeletal muscle dysfunction in the acute respiratory distress syndrome

**DOI:** 10.1186/s13054-015-0979-5

**Published:** 2015-07-02

**Authors:** D. Clark Files, Michael A. Sanchez, Peter E. Morris

**Affiliations:** Section on Pulmonary, Critical Care, Allergy and Immunologic Diseases, Wake Forest School of Medicine, Medical Center Boulevard, Winston-Salem, NC 27157 USA; Critical Illness Injury and Recovery Research Center Chadwick Miller MD Department of Emergency Medicine, Wake Forest School of Medicine, Medical Center Boulevard, Winston-Salem, NC 27157 USA

## Abstract

Patients with acute respiratory distress syndrome (ARDS) often develop severe diaphragmatic and limb skeletal muscle dysfunction. Impaired muscle function in ARDS is associated with increased mortality, increased duration of mechanical ventilation, and functional disability in survivors. In this review, we propose that muscle dysfunction in ARDS can be categorized into an early and a late phase. These early and late phases are based on the timing in relationship to lung injury and the underlying mechanisms. The early phase occurs temporally with the onset of lung injury, is driven by inflammation and disuse, and is marked predominantly by muscle atrophy from increased protein degradation. The ubiquitin-proteasome, autophagy, and calpain-caspase pathways have all been implicated in early-phase muscle dysfunction. Late-phase muscle weakness persists in many patients despite resolution of lung injury and cessation of ongoing acute inflammation-driven muscle atrophy. The clinical characteristics and mechanisms underlying late-phase muscle dysfunction do not involve the massive protein degradation and atrophy of the early phase and may reflect a failure of the musculoskeletal system to regain homeostatic balance. Owing to these underlying mechanistic differences, therapeutic interventions for treating muscle dysfunction in ARDS may differ during the early and late phases. Here, we review clinical and translational investigations of muscle dysfunction in ARDS, placing them in the conceptual framework of the early and late phases. We hypothesize that this conceptual model will aid in the design of future mechanistic and clinical investigations of the skeletal muscle system in ARDS and other critical illnesses.

## Introduction

Improvements in general critical care and ventilator management of acute respiratory distress syndrome (ARDS) over the past four decades have led to a significant reduction in mortality, from 80 % in the initial reports to the current rate of 20 % to 30 % reported in clinical trials [1]. These trends have resulted in a growing number of ARDS patients who are ICU survivors: approximately 200,000 people per year in the United States alone [2]. Unfortunately, these patients commonly have lasting sequelae, including increased mortality [3–5], physical and cognitive impairment [6–8], and reduced quality of life [9]. With the introduction of such outcomes in clinical trials, the skeletal muscle system has been increasingly recognized as a major target organ in ARDS. Clinically apparent skeletal muscle weakness in the critically ill, termed ICU-acquired weakness (ICUAW) [10, 11], occurs in up to 60 % of patients and is independently associated with mortality [12, 13].

We propose, on the basis of observations of animal models and clinical studies, that muscle wasting in patients with ARDS can be divided into early and late phases. These phases differ in pathophysiology and potential underlying mechanisms and can be identified by their relationship to the time course of lung injury, recovery, and resolution. In this review, we will summarize major recent findings regarding clinical and mechanistic investigations into muscle wasting in ARDS and frame them in the context of the early and late phases. We propose that this conceptual framework will enhance the design of future clinical and mechanistic investigations and aid in tailoring therapies designed to treat muscle wasting in ARDS.

ARDS is the more severe end of the spectrum of diseases requiring admission to an ICU. Although the muscle-wasting response of patients with ARDS has not been explicitly compared with that of critically ill patients without ARDS (that is, sepsis), patients with ARDS appear to have a very high incidence of ICUAW (up to 60 %) [12–15]. While the animal studies offer some clues to mechanistic differences between muscle wasting in ARDS and sepsis [16], further carefully controlled human studies are needed to determine whether clinical differences exist in the muscle injury and recovery trajectories of sepsis patients with and without concomitant ARDS. For these reasons, in this review, we will focus primarily on muscle wasting in ARDS, although we feel that this paradigm may prove useful in other critical illnesses, such as sepsis.

## The diagnosis of intensive care unit-acquired weakness

Since the original report by MacFarlane and Rosenthal [17], muscle wasting associated with critical illness has been called acute quadriparetic myopathy, thick filament myopathy, critical illness myopathy, critical illness polyneuropathy, and ICU-acquired paresis, among other terms. These names reflect the varying associated pathologic and electrophysiologic characteristics. The nomenclature has recently been simplified, and the term ICUAW signifies clinically measureable weakness in a critically ill patient without other known precipitating factors causing nerve or muscle injury [10, 11].

The diagnosis of ICUAW is made by using either manual muscle testing (MMT) or grip strength meters and by using specified cutoff values to denote weakness. Unfortunately, MMT is effort-dependent and insensitive and likely under-represents the degree of muscle dysfunction present in these patients [18–20]. MMT, grip strength meters, and hand-held dynamometers also all lack the ability to clearly discern muscle fatigability, which may contribute to the long-term functional impairments in ICU survivors. Other functional tests - such as the short physical performance battery [21], six-minute walk distance [8], or walk speeds [22] - may provide more information about global function, although these composite functional tests can be affected by factors other than muscle dysfunction and require a cooperative, engaged patient.

Given the limitations of these volitional measurements of muscle function in critically ill patients and survivors, other methods for identifying ICUAW are needed. Nerve conduction and direct muscle stimulation may improve the sensitivity of diagnosing ICUAW in the non-cooperative patient [23] but are infrequently used at present. Skeletal muscle ultrasound is a promising modality that can non-invasively identify the loss of muscle mass in critically ill patients; muscle echointensity values may yield additional functional information [24, 25]. These modalities remain promising, although further research is needed in this area.

Systemic ‘biomarkers’ of ICUAW would also be helpful in identifying ICUAW. Creatine phosphokinase, the most common laboratory test used for identification of myositis in other contexts, is not helpful in identifying patients with ICUAW [11, 15]. In a pilot study, peak plasma neurofilament levels were higher in patients with ICUAW, but peak levels were not reached before patients could engage in MMT, limiting the utility of this as a biomarker [26]. Another study of post-cardiac surgery patients found that insulin-like growth factor 1 (IGF-1) levels were suppressed in patients who developed ICUAW but that growth and differentiation factor 15 levels were elevated [27]. Additional studies are needed to identify systemic biomarkers that can reliably identify patients at high risk for developing ICUAW. Identifying such patients may assist in targeted allocation of physical therapy or future pharmacologic interventions.

## Phases of muscle dysfunction in acute respiratory distress syndrome

### Definition of the early phase

**Fig. 1:**
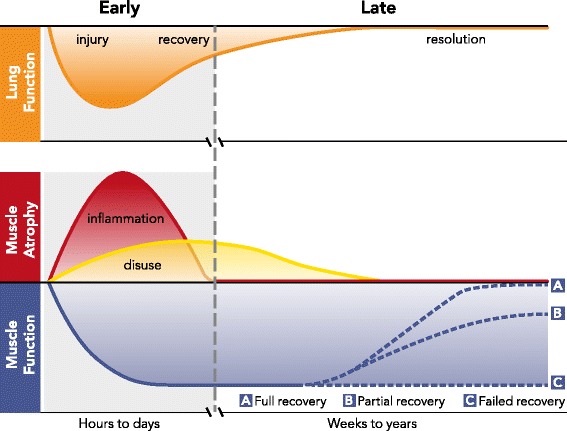
The early and late phases of muscle wasting in acute respiratory distress syndrome. The early phase of muscle wasting begins with the onset of lung injury and is caused by lung and systemic inflammation and to a lesser degree disuse, both leading to muscle atrophy. The late phase of muscle wasting begins as lung function recovers and acute systemic inflammation resolves. Disuse continues in many patients during the late phase. Muscle function deteriorates in the early phase, and dysfunction persists in many patients during the late phase, which may last for years despite resolution of lung injury and cessation of ongoing muscle atrophy. Factors mediating recovery trajectories in the late phase are poorly understood

The early phase of muscle dysfunction, which occurs hours to days after the onset of illness, begins with the activation of acute lung and systemic inflammation characteristic of early lung injury. We define the early phase to begin with the onset of the acute illness and terminate when the acute inflammation-driven muscle atrophy program resolves (Fig. 1), usually within days.

Muscle atrophy is the predominant and characteristic feature of early-phase muscle dysfunction and is driven primarily by (a) acute systemic inflammation and (b) limb and diaphragmatic muscle disuse from enforced bed rest and mechanical ventilation, respectively. Nerve, neuromuscular junction (NMJ), or direct myofiber injury or a combination of these may variably initiate atrophy or contribute to muscle weakness during the early phase. Considering the ubiquity of inflammation- and immobility-induced atrophy in these patients, we hypothesize that all patients with ARDS experience early-phase muscle wasting. We propose that atrophy is the most universal feature of ICUAW, although other pathologies such as inflammatory myopathies, polyneuropathies, or combinations also occur. Factors such as age, illness severity, organ failures, medications, malnutrition, and hypoxia may drive the severity or type of muscle dysfunction in an ancillary fashion. The drivers and clinical significance of these differing phenotypes are poorly understood. However, it is clear that both limb [12] and diaphragmatic [28, 29] muscle weakness, regardless of the pathophysiology, independently contribute to early-phase mortality.

### Definition of the late phase

The late phase of muscle dysfunction begins following resolution of the early acute lung and systemic inflammation characteristic of the early phase, usually following the first few days of illness and during the recovery phase of lung injury. Muscle atrophy may continue into the late phase, driven by disuse, but this factor usually resolves once patients are no longer bedridden. Similar to early-phase wasting, late-phase muscle weakness may occur from persistent or unresolved nerve or NMJ injury [30, 31].

The characteristic feature of late-phase muscle wasting is that muscle dysfunction persists despite recovery and resolution of lung injury in many patients [8, 20]. Factors such as age, baseline (pre-ARDS) muscle function, medications administered during or after the ICU stay, comorbidities, route of muscle injury (nerve versus NMJ versus myofiber), and nutrition may contribute to both the degree of injury and the rate of muscle functional recovery. However, the clinical characteristics associated with complete, partial, or failed recovery of muscle function in ARDS survivors (Fig. 1) are generally poorly understood.

One fundamental question is whether the recovery of muscle function in the late phase is associated with recovery of muscle mass or alternately whether weakness persists despite recovery of muscle mass. Answering this question would clarify potential mechanisms underlying persistent late-phase weakness. Unfortunately, since pre-hospital functional status of these patients is almost always unknown, it is difficult to know how baseline muscle function contributes to long-term functional outcomes. In many patients, the ‘failure to recover’ may reflect their baseline functional status pre-ARDS.

Prolonged metabolic disturbances and immune suppression have been described in survivors of burns [32] and sepsis [33]. The term post-intensive care syndrome has been used to refer to the constellation of psychiatric, cognitive, and physical function problems present in ICU survivors, including those with ARDS [34]. The relationship of systemic immunosuppression or hypermetabolism to late-phase skeletal muscle dysfunction in patients with ARDS deserves further attention.

### Pharmacologic and nutritional contributions to early- and late-phase muscle wasting

Some of the earliest reports of muscle weakness in critically ill patients associated the presence of what is now called ICUAW with both glucocorticoids and neuromuscular blockade (NMB) [35, 36]. However, more current evidence suggests that glucocorticoids, but not NMB, is associated with ICUAW [20, 23, 37]. In the most compelling recent evidence, a randomized controlled trial of the neuromuscular blocker cisatracurium for severe ARDS, the incidence of ICUAW, measured by MMT, at hospital discharge was no different from control [38].

The association of ICUAW with glucocorticoids appears stronger than that of NMB. Increased duration of glucocorticoid use is independently associated with increased myosin degradation in the skeletal muscles of critically ill patients on mechanical ventilation [39]. In the ARDS Network Long Term Outcomes study, which followed ARDS survivors enrolled in ARDS network trials, both dose of corticosteroid and ICU length of stay were associated with reduced functional outcomes at 6 and 12 months [20]. These results suggest that drugs or interventions in the ICU, even administered for short durations, can impact long-term outcomes. Other data supporting the importance of glucocorticoids in muscle wasting in ARDS include the fact that the glucocorticoid receptor is an upstream modulator of muscle ring finger 1 (MuRF1) activation [40], an important contributor to early-phase muscle wasting (see ‘The ubiquitin-proteasome system and muscle ring finger 1’ section). Overall, the available data suggest that both endogenous and exogenous glucocorticoids contribute to muscle dysfunction in ARDS.

The role of nutrition in muscle weakness in critical illness and its contribution to muscle wasting is controversial, although recent evidence suggests that increased caloric intake during the early phase does not prevent late-phase muscle dysfunction. In the long-term follow-up of patients with ARDS in the EDEN (early versus delayed enteral nutrition) trial, muscle functional outcomes were unchanged between the two arms at 6 and 12 months [6]. Emerging evidence suggests that early parenteral nutrition (PN) is detrimental for muscle function in these patients [41]. The currently available data suggest that early and full caloric nutrition, either enteral [42] or parenteral [41], does not reduce the incidence of ICUAW in critically ill patients, although future investigation is warranted. Nutritional factors may be more important for improving muscle mass when administered during the late phase.

## Early- and late-phase muscle dysfunction in acute respiratory distress syndrome: underlying mechanisms

### Mechanisms of early-phase muscle wasting

As mentioned above, the cardinal feature of early-phase muscle dysfunction is atrophy, driven by inflammation and disuse. The net balance of protein synthesis and degradation determines myofiber size. Therefore, atrophy can occur through increased protein degradation, reduced protein synthesis, or both. In most experimental models of muscle atrophy, increased muscle protein degradation - not reduced protein synthesis - accounts for the loss of muscle mass [43], although some controversy remains [44]. With regard to ARDS-associated muscle dysfunction, both increased protein degradation and reduced protein synthesis contribute to early-phase atrophy, although the former mechanism predominates. In the largest recent study measuring protein synthesis and degradation in critically ill patients (which included, but was not limited to, patients with ARDS), rectus femoris cross-sectional area decreased by 18 % over 10 days. In this study, patients in the early phase (day 1) showed reduced protein synthetic rates compared with fasted controls. At this time point, muscle protein degradation predominated over protein synthesis. By day 7, protein synthetic rate had increased compared with day 1 and fasted controls, likely an attempt of the muscle to recover from the massive protein degradation and atrophy during the inflammation-driven early phase, although the balance remained favoring ongoing atrophy [45].

In recent years, three major pathways have emerged as the primary regulators of muscle atrophy: the calpain-caspase system, the ubiquitin-proteasome system (UPS), and the autophagy-lysosome system (autophagy) [43, 46]. All have been implicated in inflammation and disuse atrophy, but their relative contributions and inter-relationships during the early phase of muscle wasting in ARDS remain incompletely understood.

#### Inflammation-driven atrophy

Both pro- and anti-inflammatory cytokines are present in the lungs and plasma of patients with ARDS [47]. Many of these pro-inflammatory cytokines are associated with muscle atrophy in humans and rodents, including tumor necrosis factor-alpha [48], interleukin (IL)-6 [49], IL-1β, and others [50, 51]. Muscle atrophy occurring via inflammatory cytokines classically requires activation of the transcription factor NF-κB (nuclear factor kappa light chain enhancer of activated B cells) [52–54], which in turn can increase muscle protein degradation, leading to rapid limb and respiratory muscle myofiber atrophy.

In lung-injured mice, marked early muscle atrophy occurs along with lung inflammation [16]. NF-κB activation in skeletal muscle is necessary for initiating the muscle atrophy during this early phase [55]. These data suggest that systemic mediators, such as inflammatory cytokines or other soluble factors that activate NF-κB, are important in the early phase of muscle atrophy in ARDS. These muscle proteolytic pathways may exist in order to provide nutritional substrates to an organism under major stress, such as massive infection or injury. In addition to promoting muscle protein degradation, pro-inflammatory cytokines may promote atrophy through inhibition of the pro-hypertrophy IGF-1/AKT pathway [56], although this concept has received less attention.

#### Disuse-driven atrophy

There is little doubt that disuse contributes to the limb muscle atrophy associated with ARDS, given the profound limb and diaphragm disuse that characterizes these patients. In fact, recent work suggests that bed rest may ‘prime’ skeletal muscle for atrophy by increasing the expression of muscle surface TLR4 (Toll-like receptor 4) receptors, which, when activated, can promote atrophy [57, 58].

However, both animal models and human data support the concept that muscle wasting associated with lung injury is phenotypically different from that induced by immobility alone. A recent report of healthy persons confined to bed rest for one week documented a 4 % loss of lean body mass [57]. In a study of critically ill patients on mechanical ventilation, muscle mass loss was approximately 12 % [45] over that same time period. Likewise, in an animal model of hind-limb immobilization, an approximately 5 % muscle mass loss of the tibialis anterior muscle was seen at day 3.5 [59], and we find an approximately 22 % muscle mass loss in the tibialis anterior of lung-injured mice at this time point [16]. Collectively, these data support the concept that disuse atrophy contributes to the early phase of wasting, but less so than inflammation-driven atrophy.

#### Molecular targets for attenuating muscle atrophy in the early phase

##### The ubiquitin-proteasome system and muscle ring finger 1

Animal models and emerging human data suggest that the UPS plays a prominent role in the early phase of limb and diaphragmatic muscle wasting in ARDS. We and others have shown that the UPS-mediated atrophy is prominent in the early phase of muscle wasting in lung-injured mice [16, 55, 60]. The E3 ligase MuRF1, which coordinates the ubiquitination of myosin heavy chain (MyHC) and other contractile proteins for proteasomal degradation [61], is necessary for early-phase atrophy in this model. Support for the importance of this mechanism in ICUAW is the finding that selective MyHC degradation is a salient pathologic feature of critical illness myopathy [62]. Others have shown that 20S proteasome activity is upregulated in the vastus lateralis of patients on mechanical ventilation, which was also associated with upregulation of the forkhead box o (FoxO) transcription factors, MuRF1, and other atrophy-promoting genes [39]. In recent work evaluating serial biopsies in mechanically ventilated patients, the only consistent change in protein expression was in MuRF1 and atrogin 1 expression, both of which were downregulated over time [45], supporting the observation that this pathway is activated in the early phase. Another study reported reduced MuRF1 levels in the muscles of critically ill patients, although the varying time points for muscle biopsies limit the interpretation of this finding [63]. The currently available human and animal data suggest that the UPS plays a prominent role in the early phase of muscle atrophy in ARDS. As therapeutic agents targeting proteins involved in UPS-mediated atrophy are developed and tested [64], their use in the early phase of ARDS-associated muscle wasting should be considered.

##### Autophagy

Briefly, macroautophagy (autophagy) is a ubiquitous process present in multiple cell types in which cellular proteins and cytoplasm are degraded and recycled via lysosomes. A focus on autophagy in skeletal muscle is relatively underexplored [65]. Increased autophagic flux can cause atrophy, although inhibition of autophagic flux can also induce atrophy, potentially through upregulation of the UPS [65, 66]. Interestingly, both the UPS and autophagy pathways can be regulated by the same FoxO transcription factors [67].

Evidence suggests that autophagy is involved in ARDS-associated muscle wasting. Diaphragmatic disuse due to mechanical ventilation in brain-dead humans is associated with the rapid appearance of autophagosomes and autophagy-related genes and proteins [68]. This finding could be due to either increased flux or a block in distal autophagy processing. In a pig model (combining mechanical ventilation, endotoxin, NMB, and corticosteroids), significant limb muscle atrophy was associated with reduction in critical autophagy genes and proteins [69].

In a prospective study of 600 patients in the EPaNIC (Early Parenteral Nutrition Completing Enteral Nutrition in Adult Critically Ill Patients) trial, 122 of whom underwent muscle biopsy, those randomly assigned to late PN had a reduced incidence of ICUAW compared with those with early PN; this result was associated with an increased LC3II-to-LC3I ratio, a marker of autophagosome formation [41]. These data suggest that autophagy induction is associated with improved muscle function.

The role of autophagy during the early phase of muscle wasting in ARDS is complex, given that either accelerated or impaired autophagy may be deleterious to muscle function. Details regarding the role of autophagy and its relationship with the UPS are still emerging, and more work is needed to determine the role of autophagy in the early phase of muscle wasting in ARDS. Other types of muscle autophagy, including microautophagy [70] and chaperone-mediated autophagy [71], also deserve future investigation in this context.

##### Caspases and calpains

Caspases and calpains are early mediators in the breakdown of sarcomeric proteins that can then undergo degradation by the UPS or autophagy pathway. Caspases and calpains have been investigated more extensively in both endotoxin- and mechanical ventilation-induced diaphragmatic dysfunction but not (to our knowledge) in animal models of lung injury. Supinski and colleagues [72] showed that calpain, caspase, and proteasome activity are upregulated in the diaphragm of endotoxin-treated mice. Likewise, diaphragm calpain activation peaks early (24 h) in the cecal ligation mouse model of sepsis. Co-administration of eicosapentoic acid prevented the loss of specific force-generating capacity in the diaphragm and prevented calpain activation [73, 74]. Others have shown that mechanical ventilation in humans causes atrophy and increased caspase 9 activity in diaphragm fibers [75]. As such, calpains and caspases remain attractive potential targets for intervention in the early phase of muscle wasting.

#### Neuropathy and other pathologies as potential therapeutic targets in the early phase

As mentioned above, polyneuropathy is found in a subset of patients with ICUAW. Critical illness polyneuropathy affects distal axonal sensory and motor nerves, which may lead to myofiber atrophy and contribute to weakness independent of atrophy. Histologically, peripheral nerves with [76, 77] or without [78] axonal degeneration have been described. The polyneuropathy in patients without nerve degeneration has been proposed to be due to a transient negative shift in voltage dependence of sodium channel fast inactivation leading to reduced excitability of the nerve, demonstrated in both rats and humans [79].

Autonomic dysregulation, which may be present in many patients with severe critical illness, may also contribute to polyneuropathy [80]. With this in mind, there has been recent interest in using β-blockade in patients with septic shock [81] as a way to attenuate sympathetic over-activation. Interestingly, stimulation of skeletal muscle β receptors leads to muscle hypertrophy through stimulating protein synthesis [82]. Therefore, muscle function should be incorporated into clinical trial design of future investigations of β-blockade in critical illness.

Epineurial and endneurial vascular leak [83] causing nerve edema is another proposed mechanism. Hyperglycemia, often characteristic of severe critical illness, could further impair nerve or muscle microcirculation [84]. This hypothesis may explain why intensive insulin therapy has been associated with a reduced incidence of ICUAW [85, 86]. Interestingly, the glucose transporter-4 (GLUT4) receptor, which modulates glucose uptake into muscle, appears mislocalized in patients with critical illness myopathy [87].

Additionally, reduced muscle membrane excitability is a common finding on electromyographic studies [23]. A series of studies has shown impaired sarcoplasmic reticulum calcium handling and impaired sodium channels in muscles of denervated and steroid-treated rodents [88–90], but to our knowledge this has not been studied in the context of lung injury. Owing to altered metabolism or increased muscle fatigue, muscle mitochondrial injury [91, 92] sustained during the early phase may contribute to muscle dysfunction.

#### Molecular targets for attenuating muscle atrophy in the late phase

Ongoing active muscle proteolysis through increased protein degradation does not appear to be a major contributing factor of weakness during the late phase. The massive inflammation-induced protein degradation has subsided at this time point [16, 45]. Therefore, therapies directed at attenuating muscle proteolysis are less likely to benefit as much as when administered during the early phase.

In contrast, enhancing protein synthesis may be useful during the late phase. Two studies suggest that there is actually already increased protein synthesis in the late phase. One study showed muscle activation of the pro-synthesis AKT-mTOR-S6k (AKT-mammalian target of rapamycin-ribosomal protein S6 kinase) pathway of critically ill patients from muscle biopsies that were obtained predominantly in the late phase [63]; a second study showed increased protein synthesis in the muscles of critically ill patients at day 7 [45]. This may be a compensatory mechanism to recover from the early phase, and studies are needed to determine whether augmenting protein synthesis pathways can improve muscle mass during the late phase. Therefore, we propose that late-phase therapies to improve muscle mass focus on enhancing protein synthesis or other factors to enhance myofiber size, such as through the myostatin pathway [93].

#### Neuropathy and other pathologies as potential therapeutic targets in the late phase

Evidence suggests that denervation injury may persist into the late phase. In a cohort of mechanically ventilated critically ill patients, muscle biopsies at about day 12 revealed upregulation of the muscle acetylcholine receptor γ mRNA, a marker of muscle denervation [39]. Late-phase wasting may also exist due to persistence of some factors initiated during the early phase, such as disuse, nerve or NMJ injury, excitation contraction uncoupling, inflammatory myopathy, or mitochondrial dysfunction.

Additional targets during the late phase include enhancing muscle regeneration by targeting muscle stem (satellite) cell activation/repair [94]. Additionally, enhancing autophagy, as a way to ‘clean up’ the misfolded proteins and other debris that accumulated during the early phase, may theoretically benefit.

**Fig. 2:**
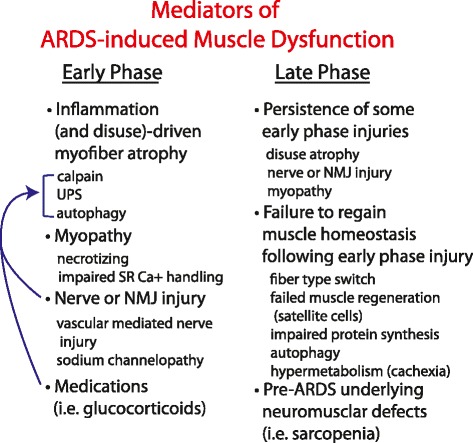
Mediators of acute respiratory distress syndrome (ARDS)-induced muscle dysfunction. Skeletal muscle atrophy is the most universal feature of the early phase, which is driven fundamentally by inflammation and disuse. Other factors such as neuropathic injury and medications can exacerbate atrophy (blue arrow) and independently cause muscle dysfunction. Therefore, inhibiting muscle protein degradation is the most promising potential early-phase therapy. The late phase is marked by cessation of inflammation-induced muscle proteolysis and therefore potential treatments at this time point will differ. Mediators of the late phase may involve persistence of some early-phase injuries or a failure to regain muscle homeostasis following the early phase. Late-phase dysfunction may be compounded by underlying pre-ARDS neuromuscular defects. NMJ, neuromuscular junction; SR Ca^+^, sarcoplasmic reticulum calcium; UPS, ubiquitin-proteasome system

Many questions remain about the relationship of the early phase to the late phase. For instance, is late-phase wasting due to persistent injuries sustained in the early phase or are the two phases mechanistically independent? Is late-phase wasting purely a reflection of a return to a pre-hospital level of reduced muscle function in patients with underlying neuromyopathies or sarcopenia? Answering these questions will clarify potential therapies to improve muscle function in ARDS survivors. Figure 2 illustrates potential clinical factors and mechanisms associated with early- and late-phase muscle wasting in ARDS.

## Currently available therapeutic approaches

Insulin administration and tight glycemic control appear to reduce ICUAW [85], although this approach has been tempered with the results of the NICE-SUGAR (Normoglycemia in Intensive Care Evaluation and Surviving Using Glucose Algorithm Regulation) trial, which suggested an increased risk of death in the tight glycemic control arm, possibly due to hypoglycemia [95]. Perhaps strategies that reduce hyperglycemia without the risk of hypoglycemia will reduce the incidence of ICUAW.

Currently, early mobilization/rehabilitation is the most readily available therapy for the attenuation of ICUAW. Evidence has demonstrated that early rehabilitation of critically ill patients is safe and has the benefit of improving other outcomes in addition to muscle strength [96–99]. Emerging evidence suggests that passive loading of the leg in a rat model of mechanical ventilation and paralysis prevented atrophy and degradation of myosin [100]. In a small study of mechanically ventilated critically ill patients, passive movement of the leg attenuated loss of specific force (but not atrophy) measured by single-fiber contraction [101]. We have recently shown that a model of early mobilization in lung-injured mice attenuates the MuRF1-mediated loss of muscle mass and force during the early phase, through an NF-κB-mediated mechanism [102]. This suggests that early mobility may attenuate the inflammation-induced atrophy in the early phase. As such, early mobilization (even passive movement) remains the best available therapy for critically ill patients to attenuate early- and late-phase muscle wasting in ARDS. Unfortunately, despite evidence that early mobility is safe and effective, there are limitations to its adoption, and implementation worldwide remains low [103, 104].

Neuromuscular electrical stimulation (NMES) may develop as an alternative therapy [105, 106], particularly for those who cannot participate in active physical therapy. In a small study, NMES attenuated type 2 myofiber atrophy, which was associated with relocalization of the GLUT4 receptor and improved glucose metabolism [87]. Further research is certainly warranted for this potential therapy.

## Conclusions

As new therapies for inhibiting muscle protein degradation become available [64], it will be critical to administer them early in critically ill patients. As we propose that muscle atrophy is the most universal feature of ICUAW and that neuropathy will also lead to downstream myofiber atrophy, therapies that attenuate muscle protein degradation during the early phase have the highest theoretical benefit to improve in-hospital and long-term outcomes. Investigators interested in the early treatment of ARDS, such as the Prevention and Early Treatment of Lung Injury (PETAL) Network, could consider approaches that aim to attenuate the early phase of muscle wasting in patients with ARDS. This approach may open a new paradigm of therapies in ARDS, a syndrome that imparts a profound and lasting effect on the musculoskeletal system.

## Abbreviations

AKT: Protein kinase B
ARDS: Acute respiratory distress syndrome
FoxO: Forkhead box o
GLUT4: Glucose transporter-4
ICUAW: Intensive care unit-acquired weakness
IGF-1: Insulin like growth factor 1
IL: Interleukin
MMT: Manual muscle testing
MuRF1: Muscle ring finger 1
MyHC: Myosin heavy chain
NF-κB: Nuclear factor kappa light chain enhancer of activated B cells
NMB: Neuromuscular blockade
NMES: Neuromuscular electrical stimulation
NMJ: Neuromuscular junction
PN: Parenteral nutrition
UPS: Ubiquitin-proteasome system
